# SARS-CoV-2 spike protein promotes inflammatory cytokine activation and aggravates rheumatoid arthritis

**DOI:** 10.1186/s12964-023-01044-0

**Published:** 2023-03-02

**Authors:** A Ram Lee, Jin Seok Woo, Seon-Yeong Lee, Yeon Su Lee, Jooyeon Jung, Chae Rim Lee, Sung-Hwan Park, Mi-La Cho

**Affiliations:** 1 Rheumatism Research Center, College of Medicine, Catholic Research Institute of Medical Science, The Catholic University of Korea, Seoul, 06591 Republic of Korea; 2 Laboratory of Translational ImmunoMedicine, Catholic Research Institute of Medical Science, College of Medicine, The Catholic University of Korea, Seoul, 06591 Korea; 3 Department of Biomedicine and Health Sciences, College of Medicine, The Catholic University of Korea, Seoul, 06591 Republic of Korea; 4 Division of Rheumatology, Department of Internal Medicine, Seoul St. Mary’s Hospital, College of Medicine, The Catholic University of Korea, Seoul, 06591 Republic of Korea; 5 Department of Medical Life Sciences, College of Medicine, The Catholic University of Korea, Seoul, 06591 Republic of Korea

**Keywords:** Coronavirus disease 2019 (COVID-19), Autoimmune disease, Rheumatoid arthritis (RA), Inflammation, Thrombosis

## Abstract

**Background:**

Coronavirus disease 2019 (COVID-19) induces inflammation, autoantibody production, and thrombosis, which are common symptoms of autoimmune diseases, including rheumatoid arthritis (RA). However, the effect of COVID-19 on autoimmune disease is not yet fully understood.

**Methods:**

This study was performed to investigate the effects of COVID-19 on the development and progression of RA using a collagen-induced arthritis (CIA) animal model. Human fibroblast-like synoviocytes (FLS) were transduced with lentivirus carrying the SARS-CoV-2 spike protein gene in vitro, and the levels of inflammatory cytokine and chemokine expression were measured. For in vivo experiments, CIA mice were injected with the gene encoding SARS-CoV-2 spike protein, and disease severity, levels of autoantibodies, thrombotic factors, and inflammatory cytokine and chemokine expression were assessed. In the in vitro experiments, the levels of inflammatory cytokine and chemokine expression were significantly increased by overexpression of SARS-CoV-2 spike protein in human FLS.

**Results:**

The incidence and severity of RA in CIA mice were slightly increased by SARS-CoV-2 spike protein in vivo. In addition, the levels of autoantibodies and thrombotic factors, such as anti-CXC chemokine ligand 4 (CXCL4, also called PF4) antibodies and anti-phospholipid antibodies were significantly increased by SARS-CoV-2 spike protein. Furthermore, tissue destruction and inflammatory cytokine level in joint tissue were markedly increased in CIA mice by SARS-CoV-2 spike protein.

**Conclusions:**

The results of the present study suggested that COVID-19 accelerates the development and progression of RA by increasing inflammation, autoantibody production, and thrombosis.

**Video Abstract**

**Supplementary Information:**

The online version contains supplementary material available at 10.1186/s12964-023-01044-0.

## Background

The severe acute respiratory syndrome coronavirus 2 (SARS-CoV-2) pandemic, also known as the coronavirus disease 2019 (COVID-19), is an unprecedented global health emergency [1]. Patients with COVID-19 commonly present with fever, cough, shortness of breath, anosmia, ageusia, and diarrhea [2]. Thrombosis has also been reported in patients with COVID-19, which is linked with higher disease severity and mortality [3, 4]. The cohort study showed that 6.68% among COVID-19 infected patients developed acute venous thromboembolism (VTE) [5]. In Sweden, it has been reported that the incidence of thrombosis was significantly increased after COVID-19 infection [6]. Thilagar et al. [7] reported the benefit of prophylactic anticoagulation for the COVID-19 infected patients. Currently, there are seven approved COVID-19 vaccines from Pfizer, AstraZeneca, Johnson & Johnson, Moderna, Sinopharm, Sinovac-CoronaVac, and Bharat Biotech. Similar to any vaccine, there have been reports of mild to moderate side effects, including fever, tiredness, muscle ache, and pain at the injection site in some cases. However, there have also been reports of thrombosis as a more serious side effect after vaccination against COVID-19. Greinacher et al. [8] reported that 11 patients presented with one or more thrombotic events, including cerebral venous thrombosis, splanchnic vein thrombosis, pulmonary embolism, and other thromboses, beginning 5–16 days after vaccination. Nine of these 11 patients were women. Schultz et al. reported five patients presenting with venous thrombosis and thrombocytopenia 7–10 days after vaccination, and four of these patients were women [9]. The mechanisms underlying these cases of post-vaccination thrombosis are unclear.

Rheumatoid arthritis (RA) is an inflammatory disease that causes long-term systemic joint inflammation [10–12]. Proinflammatory cytokines, such as interleukin (IL)-17 and IL-1β, are known to be associated with the pathogenesis of RA [13]. Many studies have shown that inflammation is associated with thrombotic factors and endothelial dysfunction in the development of atherothrombosis in patients with RA [14, 15]. In addition, recent studies have shown that patients with RA have an up to sixfold increased risk of venous thromboembolism (VTE), including deep vein thrombosis (DVT) and pulmonary embolism (PE) [16, 17].

A recent study suggested that patients with RA may have a higher risk of COVID-19 and COVID-19-related hospitalization or death compared to those without RA [18]. Patients with rheumatic diseases, particularly those taking immunosuppressive agents, may be at increased risk of severe infections. However, data regarding the severity of COVID-19 in patients with RA are still inconclusive [19–22]. Therefore, it is crucial to gain a better understanding of the mechanism underlying regulation of the development and progression of RA by COVID-19. This study was performed to examine the effects of SARS-CoV-2 on the development and progression of RA using a collagen-induced arthritis (CIA) animal model of RA.

## Methods

### Cell culture and transduction with lentivirus carrying SARS-CoV-2 spike protein

Human fibroblast-like synoviocytes (FLS) were grown in complete DMEM (12800017; Thermo Fisher Scientific, Waltham, MA, USA) supplemented with 10% (*v/v*) fetal bovine serum (FBS) (#16000044; Thermo Fisher Scientific) at 37 °C in an atmosphere of 5% CO_2_. For generation of lentivirus carrying SARS-CoV-2 spike protein [23], HEK293T cells were transfected with a plasmid encoding SARS-CoV-2 spike protein (pBOB-CAG-SARS-CoV-2-Spike-HA,#141347; Addgene, Watertown, MA, USA) and packaging vectors (pMD2.G, #12259 and psPAX2, #12260; Addgene) using Lipofectamine 2000 (#11668030; Thermo Fisher Scientific). Culture supernatants were harvested at 48 h posttransfection and aliquots of 2 × 10^5^ human FLS were transduced with the generated recombinant lentivirus. At 48 h after lentiviral transduction, cells and supernatant were harvested for qPCR and ELISA, respectively.

### RNA isolation, cDNA synthesis, and real-time quantitative PCR (qPCR)

It was performed as described previously [24]. Briefly, total mRNA from cells was collected using TRI reagent (#TR118; Molecular Research Center, Cincinnati, OH, USA). cDNA was synthesized using the Superscript Reverse Transcription system (Takara, Shiga, Japan), and real-time quantitative PCR (qPCR) was performed using LightCycler FastStart DNA Master SYBR Green I (Takara) in accordance with the manufacturer’s instructions. Expression levels were normalized relative to β-actin. The sequences of the primers used are listed in Additional file 1: Table S1.

### Enzyme-linked immunosorbent assay (ELISA)

Cell culture supernatants from the indicated cells were examined using sandwich enzyme-linked immunosorbent assay (ELISA) kits to detect the expression of IL-6 (#DY206; R&D Systems, Minneapolis, MN, USA), MCP-1 (#DY279; R&D Systems), and TNF-α (#DY210, R&D Systems). Type II collagen (CII)-specific IgG2a (A90-107P; Bethyl Laboratories, Montgomery, TX, USA) and total IgG (A90-131A; Bethyl Laboratories) in serum samples were measured using ELISA Quantitation Sets as indicated. CXCL4 (#DY595; R&D Systems) and APLA (#MBS742389; MyBioSource, San Diego, CA, USA) levels in serum samples were measured using the indicated commercial ELISA kits in accordance with the respective manufacturer’s instructions. Absorbance was measured at 405 nm on an ELISA microplate reader (Molecular Devices Inc., Sunnyvale, CA, USA).

### Collagen-induced arthritis (CIA) and spike protein injection

Male DBA1/J mice purchased from Orient Bio Inc. (Gyeonggi-do, Korea) were intradermally immunized by injection of 100 μg of chicken CII in complete Freund’s adjuvant (Chondrex Inc., Remosa, WA, USA) dissolved in 0.1 N acetic acid into the base of the tail, twice, with a 17-day interval to induce arthritis. Mice were injected intravenously with 200 μg of pLEX307-ACE2-puro (#158448; Addgene) and pcDNA3.1-SARS2-spike (#145032; Addgene) in 1 mL of saline on day 7 after the second immunization with CII. Animals in the control group were injected with the same volume of saline.

### Clinical assessment of arthritis

Arthritis score was recorded following protocol [11]. Briefly, we followed the mean arthritis index on a scale of 0–4, as follows: (0) no swelling; (1) mild swelling confined to the toes; (2) erythema and mild swelling extending from the ankle to the mid-foot; (3) moderate swelling extending from the ankle to the metatarsal joints; and (4) severe swelling encompassing the ankle, foot, and toes. Arthritis severity was determined as the sum of the scores of all legs, as assessed by two independent observers blinded to the experimental groups.

### Histopathological analysis

The animals were euthanized and the knee joints were dissected out, fixed in 10% formalin after decalcification with Decalcifying Solution-Lite (Calci-Clear Rapid; National Diagnostics, Atlanta, GA, USA), and embedded in paraffin. Tissue was cut into “Conclusions” Section μm thick with a microtome (Leica biosystems) and stained with hematoxylin and eosin (H&E).

### Immunohistochemistry

Paraffin-embedded sections were incubated at 4 °C with the following primary monoclonal antibodies: anti-IL-1β (ab9722, Abcam, Cambridge, UK), anti-TNF-α (ab6671, Abcam), anti-IL-17 (ab79056, Abcam), and anti-MCP-1 (ab7202, Abcam). The samples were next incubated with the respective secondary biotinylated antibodies, followed by incubation for 30 min with streptavidin–peroxidase complex. The reaction product was developed using 3,3′-diaminobenzidine chromogen (K3468, Dako, Carpinteria, CA, USA).

### Flow cytometry

Splenocytes were stained with the following antibodies: FITC-IFN-α (#22100-3; R&D systems), PC7-IFN-γ (#557649; BD Biosciences, Franklin Lakes, NJ, USA), APC-IL-17 (#17-7177-81; eBioscience, San Diego, CA, USA), PE-FOXP3 (#12-5773-82; eBioscience), PC5.5-CD4 (#45-0042-82; eBioscience), and APC-CD25 (#102012; BioLegend, San Diego, CA, USA). Samples were examined by flow cytometry (CytoFLEX; Beckman Coulter, Fullerton, CA, USA), and the data were analyzed using FlowJo (BD Biosciences, Franklin Lakes, NJ, USA).

### Statistical analysis

The results are shown as the mean ± standard error of the mean (SEM). Data were analyzed by Student’s *t* test or Mann–Whitney U-test using Prism 5 software (GraphPad, La Jolla, CA, USA). In all analyses, *P* < 0.05 (two-tailed) was taken to indicate statistical significance.

## Results

### Increased inflammatory cytokine and chemokine expression by SARS-CoV-2 spike protein

**Fig. 1:**
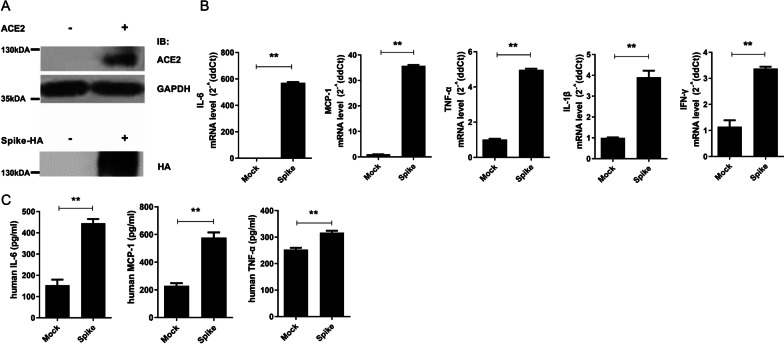
Inflammatory effect of SARS-CoV-2 spike protein in human FLS. Plasmids encoding ACE2 or SARS-CoV-2 spike protein transfected into HEK 293 cells. At 48 h after transfection, cells were harvested for immunoblot to validate their expression. **A** Immunoblot showing expression of ACE2 and SARS-CoV-2 spike protein. GAPDH was used for loading control. Human FLS were transduced with a lentivirus vector carrying SARS-CoV-2 spike protein (Spike) or empty vector (Mock). At 48 h after lentiviral transduction, cells and supernatant were harvested for qPCR and ELISA, respectively. **B** qPCR analysis of IL-6, MCP-1, TNF-α, IL-1β, and IFN-γ in the Mock and Spike groups. **C** Protein levels of IL-6, MCP-1, and TNF-α in the supernatant of Mock and Spike groups. Data are shown as the mean ± SEM. **P < 0.005 (unpaired/two-tailed *t* test)

To validate the expression of ACE2 and SARS-CoV-2 spike, we overexpressed plasmids encoding them into HEK293 cells (Fig. 1A). Human FLS cells express endogenous ACE2 receptor and its expression increases in the active RA patients [25]. To investigate the role of SARS-CoV-2 spike protein in inflammatory cytokine and chemokine expression, we overexpressed SARS-CoV-2 spike protein in human FLS. The mRNA and protein expression levels of the inflammatory cytokines, IL-6, TNF-α, IL-1β, and IFN-γ, and the chemokine, MCP-1, were markedly increased by overexpression of SARS-CoV-2 spike protein (Fig. 1B, C). These results indicated that SARS-CoV-2 induces inflammation in human FLS.

### Increased thrombosis-related marker expression by SARS-CoV-2 spike protein

**Fig. 2:**
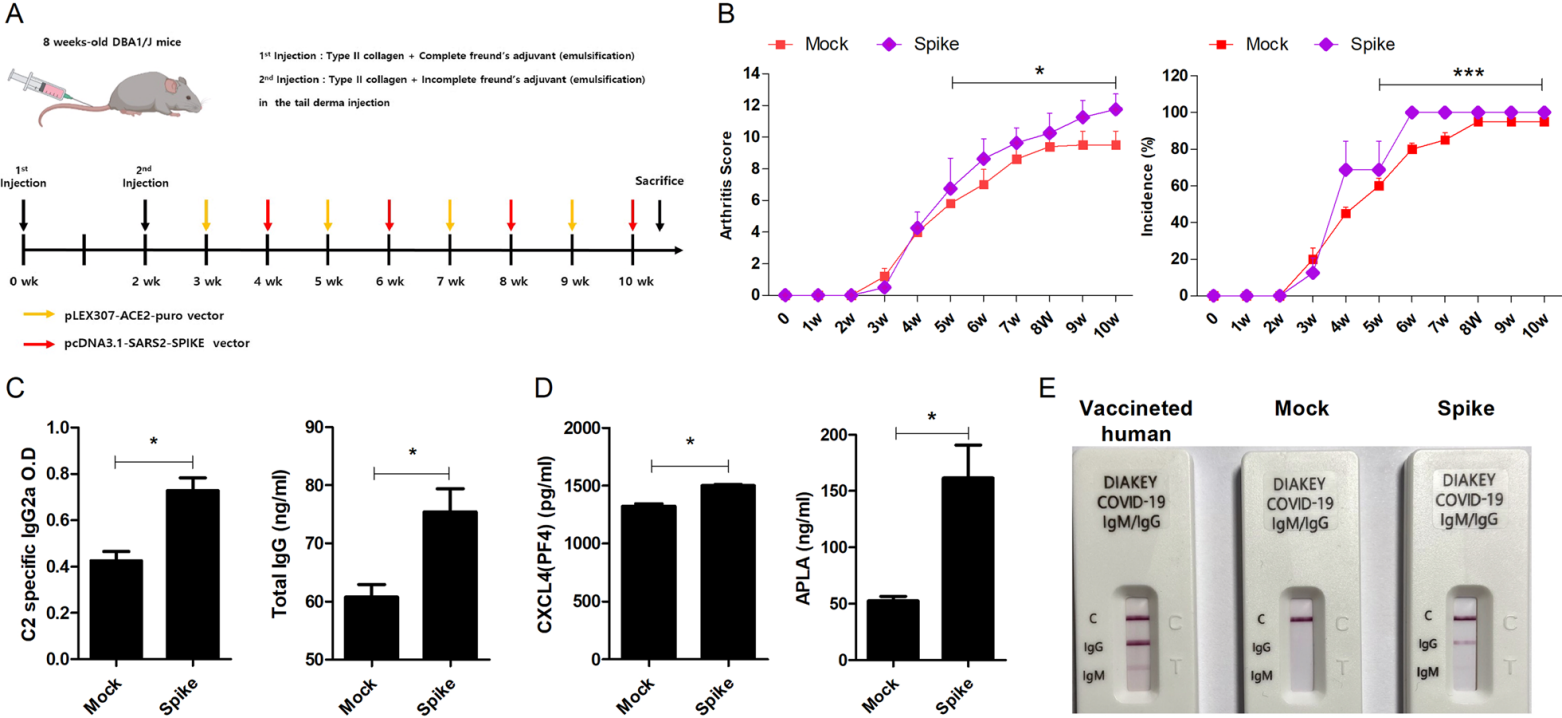
Induction of thrombosis in the RA model by SARS-CoV-2 spike protein. **A** CIA mice were euthanized at 10 weeks after injection of a plasmid encoding SARS-CoV-2 spike protein (Spike group, N = 5) or empty vector alone (Mock group, N = 5) and serum samples were collected for ELISA. **B** The arthritis scores and incidence of arthritis of CIA mice in each group are shown. **C** CII-specific IgG2a, total IgG, **D** Anti-CXCL4 autoantibody and APLA levels, **E** COVID-19 specific IgG and IgM in the serum of Mock and Spike groups. Serum of vaccinated human was used for positive control. Data are shown as the mean ± SEM from three independent experiments. *P < 0.05 (Mann Whitney U test or unpaired/two-tailed t test)

It is well known that COVID-19 is associated with thrombosis. Here, we injected CIA mice with a plasmids encoding ACE2 and SARS-CoV-2 spike protein (Spike group also known as Spike) or empty vector alone (control group also known as Mock) (Fig. 2A). The arthritis scores and incidence of arthritis were significantly increased in the Spike group compared to the control group (Fig. 2B). Next, we analyzed the levels of thrombosis-related markers, which are known to be increased in COVID-19. The ELISA results showed increasing trends of CII-specific IgG2a and total IgG in the Spike group (Fig. 2C). In addition, the levels of CXCL4 and APLA were also increased in the Spike group (Fig. 2D). We validated the effect of SARS-CoV-2 spike protein in vivo by COVID-19 test kit (Fig. 2E). We used vaccinated human sample as a positive control. These results suggested that increased thrombosis due to SARS-CoV-2 spike protein accelerates the progression of RA in the CIA mouse model.

### SARS-CoV-2 spike protein accelerates the progression of RA

**Fig. 3:**
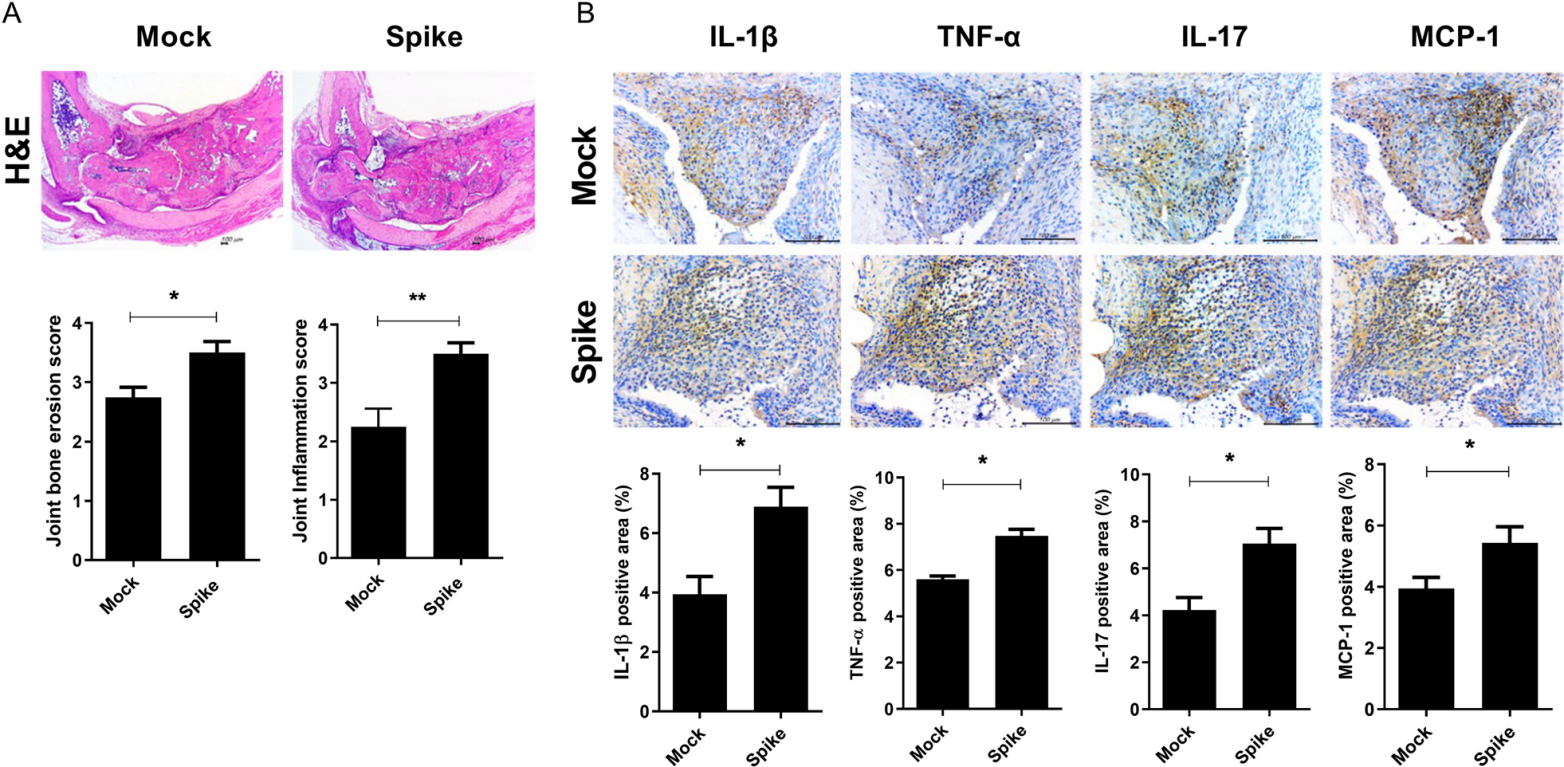
Effects of SARS-CoV-2 spike protein on the development and progression of RA. Joint tissues were collected from Control and Spike groups for histological analysis. **A** Representative images show joint tissues of mice in the Control and Spike groups stained with hematoxylin and eosin (H&E). Bar graphs show joint erosion and inflammation scores. Scale bar = 100 μm (**B**) IL-1β, TNF-α, IL-17, and MCP-1 expression in the synovium were assessed by immunohistochemistry. Bar graphs show percentages of cells labeled with each antibody. Scale bar = 100 μm, Data are shown as the mean ± SEM. *P < 0.05 (unpaired/two-tailed t test)

Next, we investigated the role of SARS-CoV-2 spike protein in the progression of RA. Mouse joint tissues were examined for tissue disruption by H&E staining (Fig. 3A). Histological analysis showed marked increases in both bone and cartilage damage and inflammation in the Spike group. Furthermore, inflammatory cytokine and chemokine level were markedly increased in the Spike group (Fig. 3B). These findings suggested that SARS-CoV-2 spike protein accelerates RA by increasing inflammatory cytokine and chemokine expression.

### Increased antiviral cytokine expression by SARS-CoV-2 spike protein

**Fig. 4:**
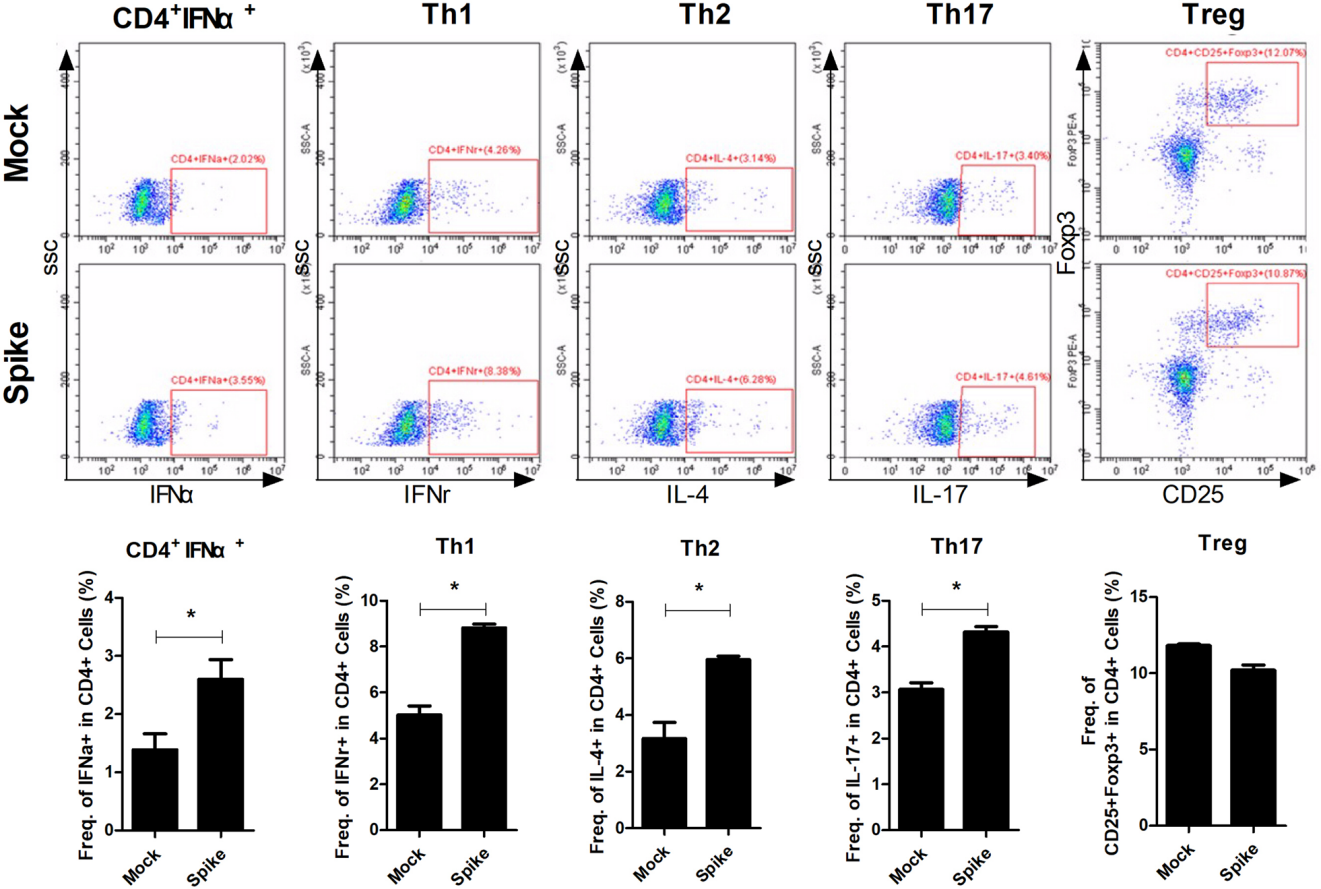
Increased type I IFN expression by SARS-CoV-2 spike protein. Splenocytes were collected from mice in the Control and Spike groups for flow cytometric analysis. The population of CD4^+^IFN-α^+^, CD4^+^IFN-γ^+^ (Th1), CD4^+^IL-4^+^ (Th2), CD4^+^IL-17^+^ (Th17), and CD4^+^CD25^+^FOXP3^+^ (Treg) cells were measured by flow cytometry in splenocytes of the Control and Spike groups. *P < 0.05 (unpaired/two-tailed *t* test)

Type I IFN expression is known to be increased by viral infection. Type I IFN is known to mainly expressed by plasmacytoid dendritic cells (pDCs) and fibroblast. Recent studies revealed that T cells express more STING than macrophage and produce type I IFN [26–28]. In this study, we found that type I IFN expression in splenocytes were markedly increased in the Spike groups (Fig. 4). Furthermore, the Th1, Th2, and Th17 population were also markedly increased in the Spike groups, while Treg population was decreased. And their expression was inhibited by STING inhibitor, H-151 (Additional file 1: Supplementary Figure 1). Type I IFN is also known to induce thrombosis. Taken together, our findings suggested that SARS-CoV-2 spike protein accelerates the development and progression of RA via its effects on the type I IFN–thrombosis axis.

## Discussion

As exposure to SARS-CoV-2 has become almost inevitable, it is essential to understand the effects of viral infection and minimize its impact. It has been reported that COVID-19 affects the severity of autoimmune diseases, including RA [29–31]. This study was performed to examine the effects of SARS-CoV-2 infection on the development and progression of RA. Here, we analyzed the severity of RA associated with SARS-CoV-2 spike protein in a CIA mouse model of RA. Our in vitro experiments showed that the expression levels of the proinflammatory cytokines, IL-6, TNF-α, IL-1β, and IFN-γ, and the chemokine, MCP-1, were increased by SARS-CoV-2 spike protein. SARS-CoV-2 infection has been reported to induce a hyperinflammatory response [32, 33]. In our in vivo experiments, severe bone erosion and joint damage were observed in mice in the Spike groups compared to the control group. In addition, the proinflammatory cytokine and chemokine level in the joint tissue were increased in the Spike group compared to the control group. The serum IL-6, IL-8, TNF-α, and IL-1β levels were reported to be elevated in hospitalized patients with COVID-19. In addition, proinflammatory cytokines, such as IL-6, IL-15, and IL-1, were shown to be correlated with the severity of disease associated with MERS-CoV, SARS-CoV, and SARS-CoV-2 infection [33, 34]. Such proinflammatory cytokines are also known to be hallmarks of RA. It has been reported that increased levels of proinflammatory cytokines induce and accelerate the development and progression of RA [35–37]. The increases in proinflammatory cytokine levels in COVID-19 may accelerate the development and progression of RA.

The levels of anti-CXCL4 (also called PF4) autoantibodies and APLA were increased in the Spike groups compared to the control group in the present study. It has been reported that autoantibodies to CXCL4 and APLA were increased in patients with RA [38–40]. These factors are known to be associated with thrombosis. Thrombosis occurs frequently in patients with severe COVID-19 and contributes significantly to mortality. Anti-phospholipid syndrome (APS) is an autoimmune clotting condition, in which individuals generate antibodies that target the tissues and cells of their own body [41]. Several studies have reported that levels of autoantibodies to CXCL4 and APLA are significantly elevated in patients with COVID-19 [42–44]. The increased levels of thrombosis-associated factors by COVID-19 in RA may accelerate disease progression.

Type I IFNs have been implicated in the pathogenesis of rheumatic diseases, including systemic lupus erythematosus (SLE), Sjögren’s syndrome, and RA [45]. Type I IFNs are mainly produced by plasmacytoid dendritic cells (pDCs) following viral infection to inhibit viral replication [46]. Type I IFNs also have immunostimulatory properties, including activation of B cells to produce autoantibodies, which are abnormal antibodies targeting the cells and tissues of the body [47]. Several recent studies have reported that autoantibody levels are elevated and thrombosis occurs in patients with COVID-19 [48, 49]. In this study, we found that the levels of type I IFNs and autoantibodies were higher in the Spike groups than the control group. Furthermore, blood clotting factors, anti-CXCL4 autoantibody and APLA, were also were higher in the Spike groups than the control group. Our data indicated that the development and progression of RA are accelerated by COVID-19 through increases in type I IFNs followed by increased levels of autoantibodies and thrombosis-associated factors.

Autoimmune diseases, including SLE, Sjögren’s syndrome, and RA, are conditions in which the immune system attacks the host’s own cells and tissue [50, 51]. These diseases are characterized by increased cytokine levels in the tissue and elevated levels of autoantibodies. In addition, these diseases are also accompanied by increased blood clotting. Several cohort studies showed that inflammatory cytokine and autoantibody levels were increased in patients with COVID-19. In addition, thrombosis has been reported to occur in COVID-19 patients. The results of the present study showed that proinflammatory cytokine and chemokine levels were increased in the Spike groups compared to the control group. Furthermore, the levels of type I IFNs, autoantibodies, and thrombosis-associated factors were also increased in the Spike groups. Finally, we found that SARS-CoV-2 spike protein increased the severity of RA in a CIA mouse model.

## Conclusions

These findings suggest that SARS-CoV-2 spike protein may accelerate the development and progression of RA by increasing the levels of proinflammatory cytokines and thrombosis-associated factors. Our results provide the insight for therapy or prevention on not only COVID-19 but also other viral infection on the patient with autoimmune diseases.

## Supplementary Information

**Additional file 1**. **Table S1**: List of primers for real-time PCR in this study. **Supplementary Figure 1.** The expression of type I IFN suppressed by STING inhibitor, H-151. Splenocyte were cultured with anti-CD3 (0.5ug/ml) in the absence or presence of H-151 (0.5 ug/ml, 5 ug/ml) for 3day. Cells and culture supernatant were harvest for flow cytometry and ELISA, respectively. **A.** The population of CD4+IFN-α+, CD4+IFN-γ+ (Th1), CD4+IL-4+ (Th2), CD4+IL-17+ (Th17), and CD4+CD25+FOXP3+ (Treg) cells were measured by flow cytometry in splenocytes. **B.** TNF-α, IL-17 and total IgG were measured in culture supernatant by ELISA. Data are shown as the mean ± SEM from three independent experiments. (Mann Whitney U test or unpaired/two-tailed t test).

## Data Availability

All data generated or analysed during this study are included in this published article.

## References

[1] Ragab D, Salah Eldin H, Taeimah M, Khattab R, Salem R (2020). The COVID-19 cytokine storm; what we know so far. Front Immunol.

[2] Wu C, Chen X, Cai Y, Xia J, Zhou X, Xu S (2020). Risk factors associated with acute respiratory distress syndrome and death in patients with coronavirus disease 2019 pneumonia in Wuhan, China. JAMA Intern Med.

[3] Middeldorp S, Coppens M, van Haaps TF, Foppen M, Vlaar AP, Muller MCA (2020). Incidence of venous thromboembolism in hospitalized patients with COVID-19. J Thromb Haemost.

[4] Klok FA, Kruip M, van der Meer NJM, Arbous MS, Gommers D, Kant KM (2020). Incidence of thrombotic complications in critically ill ICU patients with COVID-19. Thromb Res.

[5] Lee Y, Jehangir Q, Li P, Gudimella D, Mahale P, Lin CH (2022). Venous thromboembolism in COVID-19 patients and prediction model: a multicenter cohort study. BMC Infect Dis.

[6] Katsoularis I, Fonseca-Rodriguez O, Farrington P, Jerndal H, Lundevaller EH, Sund M (2022). Risks of deep vein thrombosis, pulmonary embolism, and bleeding after covid-19: nationwide self-controlled cases series and matched cohort study. BMJ.

[7] Thilagar B, Beidoun M, Rhoades R, Kaatz S (2021). COVID-19 and thrombosis: searching for evidence. Hematology Am Soc Hematol Educ Program.

[8] Greinacher A, Thiele T, Warkentin TE, Weisser K, Kyrle PA, Eichinger S (2021). Thrombotic thrombocytopenia after ChAdOx1 nCov-19 vaccination. N Engl J Med.

[9] Schultz NH, Sorvoll IH, Michelsen AE, Munthe LA, Lund-Johansen F, Ahlen MT (2021). Thrombosis and Thrombocytopenia after ChAdOx1 nCoV-19 Vaccination. N Engl J Med.

[10] Larid G, Pancarte M, Offer G, Clavel C, Martin M, Pradel V (2021). In Rheumatoid arthritis patients, HLA-DRB1*04:01 and rheumatoid nodules are associated with ACPA to a particular fibrin epitope. Front Immunol.

[11] Jhun J, Moon J, Ryu J, Shin Y, Lee S, Cho KH (2020). Liposome/gold hybrid nanoparticle encoded with CoQ10 (LGNP-CoQ10) suppressed rheumatoid arthritis via STAT3/Th17 targeting. PLoS ONE.

[12] Na HS, Lee SY, Min HK, Park WJ, Lee JH, Cho KH (2020). The establishment of a rheumatoid arthritis primate model in Macaca fascicularis. J Transl Med.

[13] Tansakul M, Thim-Uam A, Saethang T, Makjaroen J, Wongprom B, Pisitkun T (2020). Deficiency of STING promotes collagen-specific antibody production and B Cell survival in collagen-induced arthritis. Front Immunol.

[14] Shoenfeld Y, Gerli R, Doria A, Matsuura E, Cerinic MM, Ronda N (2005). Accelerated atherosclerosis in autoimmune rheumatic diseases. Circulation.

[15] Bisoendial RJ, Levi M, Tak PP, Stroes ES (2010). The prothrombotic state in rheumatoid arthritis: an additive risk factor for adverse cardiovascular events. Semin Thromb Hemost.

[16] Johannesdottir SA, Schmidt M, Horvath-Puho E, Sorensen HT (2012). Autoimmune skin and connective tissue diseases and risk of venous thromboembolism: a population-based case-control study. J Thromb Haemost.

[17] Bacani AK, Gabriel SE, Crowson CS, Heit JA, Matteson EL (2012). Noncardiac vascular disease in rheumatoid arthritis: increase in venous thromboembolic events?. Arth Rheum.

[18] England BR, Roul P, Yang Y, Kalil AC, Michaud K, Thiele GM (2021). Risk of COVID-19 in rheumatoid arthritis: a national veterans affairs matched cohort study in at-risk individuals. Arth Rheumatol.

[19] Hasseli R, Mueller-Ladner U, Hoyer BF, Krause A, Lorenz HM, Pfeil A (2021). Older age, comorbidity, glucocorticoid use and disease activity are risk factors for COVID-19 hospitalisation in patients with inflammatory rheumatic and musculoskeletal diseases. RMD Open.

[20] Marques CDL, Kakehasi AM, Pinheiro MM, Mota LMH, Albuquerque CP, Silva CR (2021). High levels of immunosuppression are related to unfavourable outcomes in hospitalised patients with rheumatic diseases and COVID-19: first results of ReumaCoV Brasil registry. RMD Open.

[21] Bachiller-Corral J, Boteanu A, Garcia-Villanueva MJ, de la Puente C, Revenga M, Diaz-Miguel MC (2021). Risk of severe COVID-19 infection in patients with inflammatory rheumatic diseases. J Rheumatol.

[22] Gianfrancesco M, Hyrich KL, Al-Adely S, Carmona L, Danila MI, Gossec L (2020). Characteristics associated with hospitalisation for COVID-19 in people with rheumatic disease: data from the COVID-19 Global Rheumatology Alliance physician-reported registry. Ann Rheum Dis.

[23] Ogawa J, Zhu W, Tonnu N, Singer O, Hunter T, Ryan AL, et al. The D614G mutation in the SARS-CoV2 Spike protein increases infectivity in an ACE2 receptor dependent manner. bioRxiv. 2020.

[24] Kim EK, Min HK, Lee SY, Kim DS, Ryu JG, Na HS (2020). Metformin rescues rapamycin-induced mitochondrial dysfunction and attenuates rheumatoid arthritis with metabolic syndrome. Arthritis Res Ther.

[25] Mokuda S, Tokunaga T, Masumoto J, Sugiyama E (2020). Angiotensin-converting enzyme 2, a SARS-CoV-2 receptor, is upregulated by Interleukin 6 through STAT3 signaling in synovial tissues. J Rheumatol.

[26] Larkin B, Ilyukha V, Sorokin M, Buzdin A, Vannier E, Poltorak A (2017). Cutting edge: activation of STING in T cells induces type I IFN responses and cell death. J Immunol.

[27] Gulen MF, Koch U, Haag SM, Schuler F, Apetoh L, Villunger A (2017). Signalling strength determines proapoptotic functions of STING. Nat Commun.

[28] Imanishi T, Unno M, Kobayashi W, Yoneda N, Matsuda S, Ikeda K (2019). Reciprocal regulation of STING and TCR signaling by mTORC1 for T-cell activation and function. Life Sci Alliance.

[29] Liu Y, Sawalha AH, Lu Q (2021). COVID-19 and autoimmune diseases. Curr Opin Rheumatol.

[30] Tan EH, Sena AG, Prats-Uribe A, You SC, Ahmed WU, Kostka K (2021). COVID-19 in patients with autoimmune diseases: characteristics and outcomes in a multinational network of cohorts across three countries. Rheumatology.

[31] Galeotti C, Bayry J (2020). Autoimmune and inflammatory diseases following COVID-19. Nat Rev Rheumatol.

[32] Del Valle DM, Kim-Schulze S, Huang HH, Beckmann ND, Nirenberg S, Wang B (2020). An inflammatory cytokine signature predicts COVID-19 severity and survival. Nat Med.

[33] Pacha O, Sallman MA, Evans SE (2020). COVID-19: a case for inhibiting IL-17?. Nat Rev Immunol.

[34] Mahallawi WH, Khabour OF, Zhang Q, Makhdoum HM, Suliman BA (2018). MERS-CoV infection in humans is associated with a pro-inflammatory Th1 and Th17 cytokine profile. Cytokine.

[35] Robert M, Miossec P (2018). IL-17 in rheumatoid arthritis and precision medicine: from synovitis expression to circulating bioactive levels. Front Med.

[36] Farrugia M, Baron B (2016). The role of TNF-alpha in rheumatoid arthritis: a focus on regulatory T cells. J Clin Transl Res.

[37] Mehta S, Akhtar S, Porter RM, Onnerfjord P, Bajpayee AG (2019). Interleukin-1 receptor antagonist (IL-1Ra) is more effective in suppressing cytokine-induced catabolism in cartilage-synovium co-culture than in cartilage monoculture. Arthritis Res Ther.

[38] Yeo L, Adlard N, Biehl M, Juarez M, Smallie T, Snow M (2016). Expression of chemokines CXCL4 and CXCL7 by synovial macrophages defines an early stage of rheumatoid arthritis. Ann Rheum Dis.

[39] Marziale A, Bettacchioli E, Picart G, Nafai S, Galinat H, Meroni PL (2020). Antiphospholipid autoantibody detection is important in all patients with systemic autoimmune diseases. J Autoimmun.

[40] van Delft MAM, Huizinga TWJ (2020). An overview of autoantibodies in rheumatoid arthritis. J Autoimmun.

[41] Ludwig RJ, Vanhoorelbeke K, Leypoldt F, Kaya Z, Bieber K, McLachlan SM (2017). Mechanisms of autoantibody-induced pathology. Front Immunol.

[42] Zhang S, Liu Y, Wang X, Yang L, Li H, Wang Y (2020). SARS-CoV-2 binds platelet ACE2 to enhance thrombosis in COVID-19. J Hematol Oncol.

[43] Tung ML, Tan B, Cherian R, Chandra B (2021). Anti-phospholipid syndrome and COVID-19 thrombosis: connecting the dots. Rheumatol Adv Pract.

[44] Wang EY, Mao T, Klein J, Dai Y, Huck JD, Jaycox JR (2021). Diverse functional autoantibodies in patients with COVID-19. Nature.

[45] Di Domizio J, Cao W (2013). Fueling autoimmunity: type I interferon in autoimmune diseases. Expert Rev Clin Immunol.

[46] Hall JC, Rosen A (2010). Type I interferons: crucial participants in disease amplification in autoimmunity. Nat Rev Rheumatol.

[47] Xiao ZX, Miller JS, Zheng SG (2021). An updated advance of autoantibodies in autoimmune diseases. Autoimmun Rev.

[48] Ronit A, Jorgensen SE, Roed C, Eriksson R, Iepsen UW, Plovsing RR (2021). Host genetics and antiviral immune responses in adult patients with multisystem inflammatory syndrome. Front Immunol.

[49] van der Wijst MGP, Vazquez SE, Hartoularos GC, Bastard P, Grant T, Bueno R (2021). Type I interferon autoantibodies are associated with systemic immune alterations in patients with COVID-19. Sci Transl Med.

[50] Hong SM, Lee J, Jang SG, Lee J, Cho ML, Kwok SK (2020). Type I interferon increases inflammasomes associated pyroptosis in the salivary glands of patients with primary sjogren's syndrome. Immune Netw.

[51] Park CS, Kim SH, Lee CK (2020). Immunotherapy of autoimmune diseases with nonantibiotic properties of tetracyclines. Immune Netw.

