# Supplementary material for: SARS-CoV-2 spike protein promotes inflammatory cytokine activation and aggravates rheumatoid arthritis
Source: Cell Commun Signal. 2023 Mar 2;21:44. doi: 10.1186/s12964-023-01044-0 (PMC9978284; doi:10.1186/s12964-023-01044-0)
Supplement: Supplementary file 2 — Additional file 1. Table S1: List of primers for real-time PCR in this study. Supplementary Figure 1. The expression of type I IFN suppressed by STING inhibitor, H-151. Splenocyte were cultured with anti-CD3 (0.5ug/ml) in the absence or presence of H-151 (0.5 ug/ml, 5 ug/ml) for 3day. Cells and culture supernatant were harvest for flow cytometry and ELISA, respectively. A. The population of CD4+IFN-α+, CD4+IFN-γ+ (Th1), CD4+IL-4+ (Th2), CD4+IL-17+ (Th17), and CD4+CD25+FOXP3+ (Treg) cells were measured by flow cytometry in splenocytes. B. TNF-α, IL-17 and total IgG were measured in culture supernatant by ELISA. Data are shown as the mean ± SEM from three independent experiments. (Mann Whitney U test or unpaired/two-tailed t test). [file 12964_2023_1044_MOESM2_ESM.docx]

# SARS-CoV-2 spike protein promotes inflammatory cytokine activation and aggravates rheumatoid arthritis

A Ram Lee^1,2,3,*^, Jin Seok Woo^1,2,*^, Seon-Yeong Lee^1,2^, Yeon Su Lee^1,2,3^, Jooyeon Jung^1,2^, Chae Rim Lee^1,2,3^ ,Sung-Hwan Park^4, #^, Mi-La Cho^1, 2,3,5, #^

^1^ Rheumatism Research Center, College of Medicine, Catholic Research Institute of Medical Science, The Catholic University of Korea, Seoul 06591, Republic of Korea

^2^ Lab of Translational ImmunoMedicine, Catholic Research Institute of Medical Science, College of Medicine, The Catholic University of Korea, Seoul 06591, Korea

^3^ Department of Biomedicine & Health Sciences, College of Medicine, The Catholic University of Korea, Seoul 06591, Republic of Korea

^4^ Division of Rheumatology, Department of Internal Medicine, Seoul St. Mary's Hospital, College of Medicine, The Catholic University of Korea, Seoul 06591, Republic of Korea

^5^ Department of Medical Life Sciences, College of Medicine, The Catholic University of Korea, Seoul 06591, Republic of Korea

* These authors have contributed equally to this work.

^#^ Authors to whom correspondence should be addressed. Email: rapark@catholic.ac.kr (S.H.P.), iammila@catholic.ac.kr (M.L.C.)

**Correspondence should be addressed to**

**Mi-La Cho, PhD,** Rheumatism Research Center, Catholic Research Institute of Medical Science, College of Medicine, The Catholic University of Korea, Seoul 06591, Republic of Korea (Tel: 82-2-2258-7473, Fax: 82-2-2258-7473, E-mail: [iammila@catholic.ac.kr](mailto:iammila@catholic.ac.kr))**SUPPLEMENTAL INFORMATION**

**Supplementary Table**

Supplementary Table 1. List of primers for real-time PCR in this study

| **Gene name** | **Forward Primer** | **Reverse Primer** |
| --- | --- | --- |
| SARS-CoV2-Spike | CGCCGCGACTAAAATGTCTG | GCGGAAAATGAGCTTTGCCA |
| IL-6 | AATTCGGTACATCCTCGACGG | GGTTGTTTTCTGCCAGTGCC |
| MCP-1 | CAGCCAGATGCAATCAATGC | GTGGTCCATGGAATCCTGAA |
| TNF-α | GCCTCTTCTCCTTCCTGATCGT | CTCGGCAAAGTCGAGATAGTCG |
| IL-1β | GGACAAGCTGAGGAAGATGC | TCGTTATCCCATGTGTCGAA |
| IFN-γ | GAGTGTGGAGACCATCAAGGA | TTAGCTGCTGGCGACAGTTC |
| β-actin | GGACTTCGAGCAAGAGATGG | TGTGTTGGGGTACAGGTCTTTG |


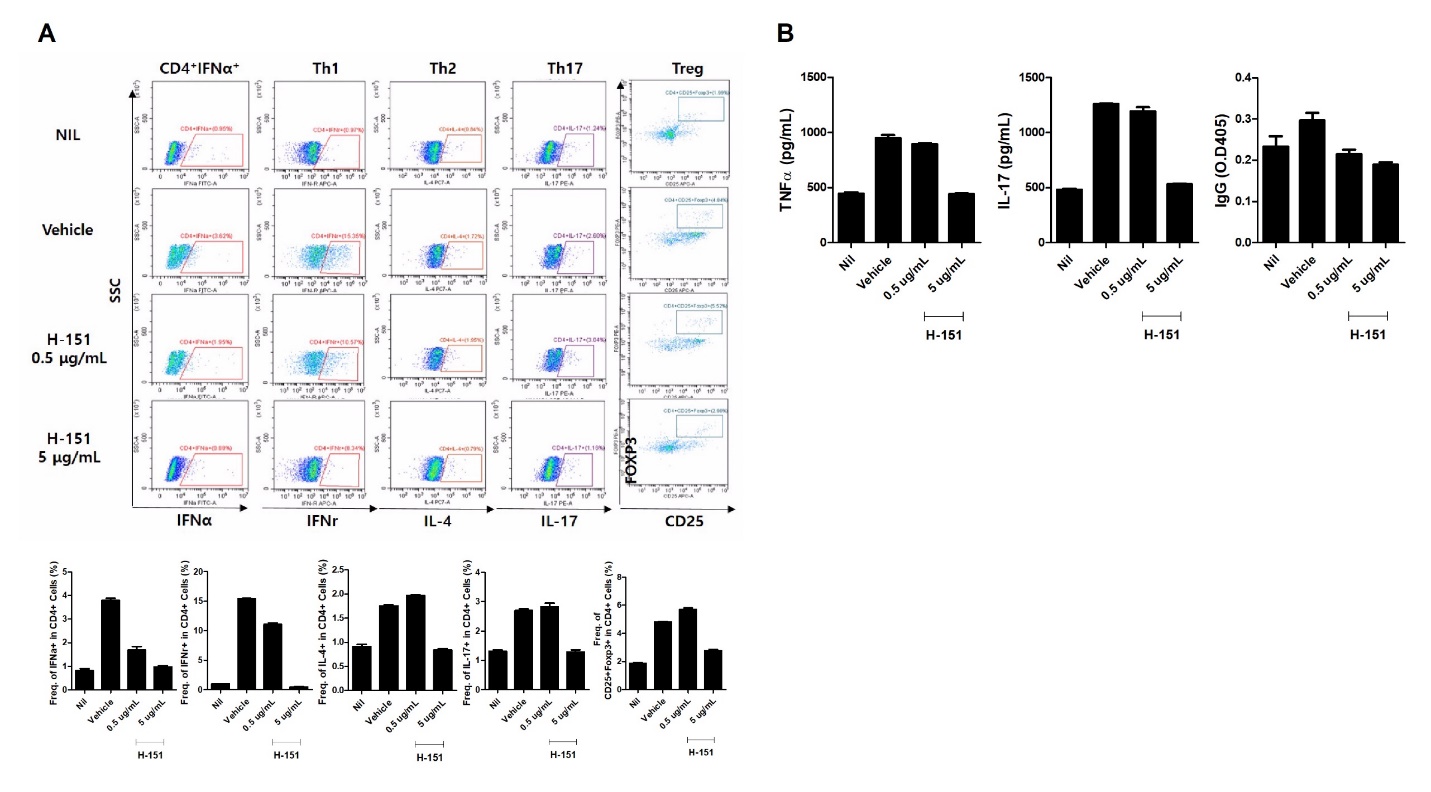


**Supplementary Figure 1. The expression of type I IFN suppressed by STING inhibitor, H-151.** Splenocyte were cultured with anti-CD3 (0.5ug/ml) in the absence or presence of H-151 (0.5 ug/ml, 5 ug/ml) for 3day. Cells and culture supernatant were harvest for flow cytometry and ELISA, respectively. **A.** The population of CD4^+^IFN-α^+^, CD4^+^IFN-γ^+^ (Th1), CD4^+^IL-4^+^ (Th2), CD4^+^IL-17^+^ (Th17), and CD4^+^CD25^+^FOXP3^+^ (Treg) cells were measured by flow cytometry in splenocytes. **B.** TNF-α, IL-17 and total IgG were measured in culture supernatant by ELISA. Data are shown as the mean ± SEM from three independent experiments. (*Mann Whitney U test or* unpaired/two-tailed t test)
